# Dopamine Neuron Challenge Test for early detection of Parkinson’s disease

**DOI:** 10.1038/s41531-021-00261-z

**Published:** 2021-12-16

**Authors:** Jingheng Zhou, Jicheng Li, Amy B. Papaneri, Nicholas P. Kobzar, Guohong Cui

**Affiliations:** In Vivo Neurobiology Group, Neurobiology Laboratory, National Institute of Environmental Health Sciences, National Institutes of Health, 111 T.W. Alexander Drive, Research Triangle Park, NC 27709 USA

**Keywords:** Parkinson's disease, Parkinson's disease

## Abstract

Diagnosing Parkinson’s disease (PD) before the clinical onset proves difficult because the hallmark PD symptoms do not manifest until more than 60% of dopamine neurons in the substantia nigra pars compacta have been lost. Here we show that, by evoking a transient dopamine release and subsequently measuring the levels of dopamine metabolites in the cerebrospinal fluid and plasma, a hypodopaminergic state can be revealed when less than 30% of dopamine neurons are lost in mouse PD models. These findings may lead to sensitive and practical screening and diagnostic tests for detecting early PD in the high-risk population.

## Introduction

Parkinson’s disease (PD) is the second most common neurodegenerative disease and the most common movement disorder, affecting 1% of the population over the age of 60^1,2^. The hallmark pathological feature of PD is the progressive loss of dopamine (DA)-producing neurons (DANs) in the substantia nigra pars compacta (SNc), resulting in a cohort of motor deficits that worsen over time^3^. Since the loss of DANs is irreversible, earlier diagnosis of PD can allow for earlier interventions^4^ to slow the disease progression and ultimately to arrest PD in the prodromal stage. However, since PD motor symptoms typically do not manifest until more than 60% of SNc DANs have been lost^5–7^, detection of the early-stage PD before the clinical onset is challenging. Though some success has been reported using neuroimaging methods, such as positron emission tomography (PET) and single-photon emission computed tomography (SPECT)-based DaTscan, to target DA transporter (DAT), these methods typically involve radiolabeled ligands and are not practical for general screening in the high-risk population^8^. The goal of this study is to develop a sensitive and practical laboratory test that can detect PD at an early stage.

**Fig. 1:**
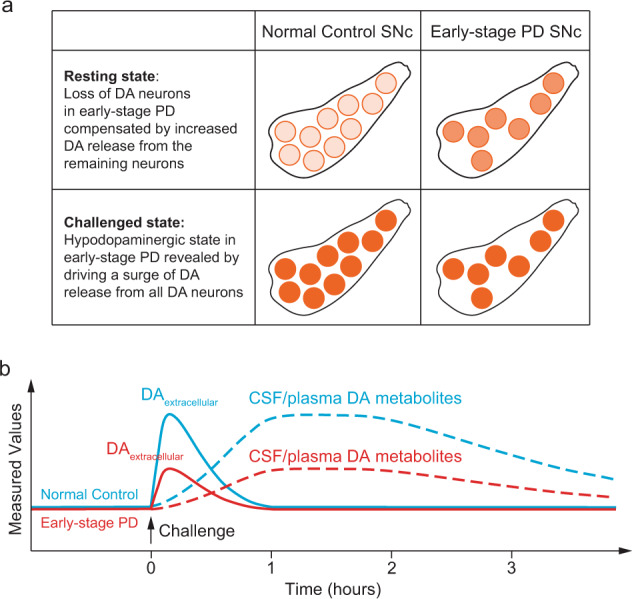
Illustration of the hypothesis underlying the “Dopamine Neuron Challenge Test” (DNC Test). **a** Schematic illustration to show the number of DANs (orange circles) and the amount of dopamine released from each DA neuron (indicated by the density of the filling color) in the SNc of normal controls (left) and early-stage PD patients (right) at the resting state (top) and after challenged by drugs that promote DA release (bottom). **b** Proposed changes in the levels of extracellular DA in the brain (solid lines) and the DA metabolites in CSF and plasma (dashed lines) in normal controls (blue) and early-stage PD patients (red) before and after the drug challenge.

We hypothesized that, at the early, prodromal stage of PD, the initial loss of DANs could be compensated by the homeostatic increase of dopamine release from the remaining DANs at the resting state (Fig. 1a). Thus, the baseline extracellular DA level may be maintained at a normal range at this stage (Fig. 1b). However, if we apply a challenge to transiently perturb the homeostasis and stimulate DA release from all DANs, the magnitude of total DA release should be smaller in PD patients because of the reduced number of DANs (Fig. 1a). We further hypothesized that the difference in the magnitude of evoked total DA release between healthy subjects and PD patients could translate to different levels of DA metabolites in the readily accessible body fluid samples, such as cerebrospinal fluid (CSF) and plasma (Fig. 1b). We have named this new method the “Dopamine Neuron Challenge Test” (DNC Test) and have tested it in two mouse PD models.

## Results

### Selection of pharmacological agents for DNC Test

**Fig. 2:**
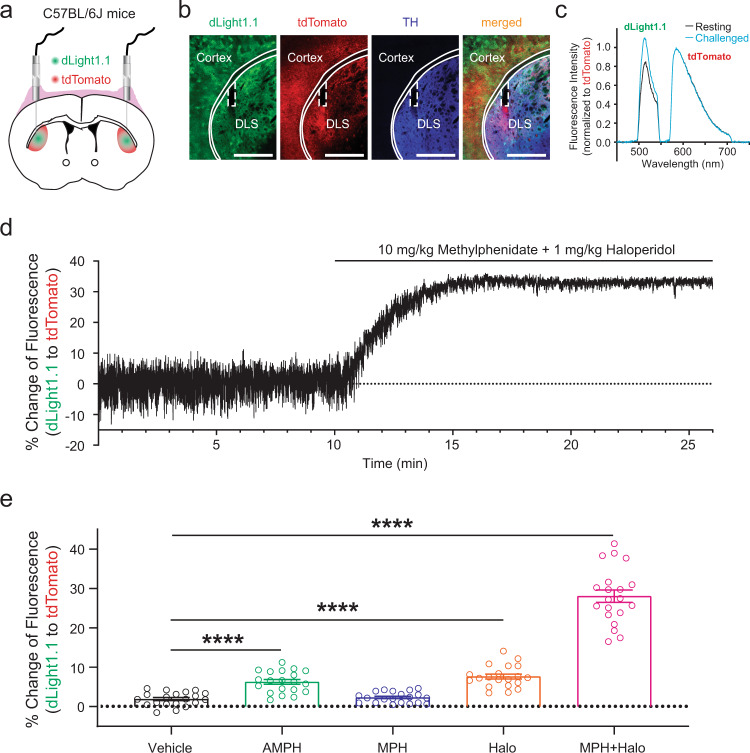
Comparison of the challenging effect between different agents on striatal extracellular dopamine level in vivo in normal C57BL/6 J mice. **a** Schematic illustration of the intrastriatal fiber photometry recordings using green fluorescent DA sensor dLight1.1 and red fluorescent protein tdTomato. **b** Immunofluorescence images to show the expression of dLight1.1, tdTomato, and TH. In this particular immunostaining experiment, TH is labelled with Alexa Fluor 647 secondary antibody and dLight1.1 is labelled with Alexa Fluor 488 secondary antibody, whereas tdTomato is unstained and visualized by its endogenous fluorescence. Scale: 1000 µm. Cortex and DLS (dorsolateral striatum) were indicated with solid line. Optical-fiber tip was indicated with dashed line. **c**, **d** Representative emission spectra of dLight1.1 and tdTomato, normalized to the tdTomato emission peak (**c**), and the dynamic changes of extracellular DA level, represented by the fluorescence ratio of dLight1.1 to tdTomato in a freely moving mouse (**d**) before and after an i.p. injection of methylphenidate (10 mg/kg) + haloperidol (1 mg/kg). **e** Comparison of the percent increase in the ratio of dLight1.1/tdTomato caused by different compounds. ****, *p* < 0.0001, one-way ANOVA followed by Dunnett’s multiple comparisons test. *n* = 20 samples recorded bilaterally from 10 C57BL/6 J mice for each group. All data are plotted as Mean ± SEM overlaid with individual replicates.

To identify a robust and safe protocol for challenging DANs, we first compared several FDA-approved drugs that are known to modulate the release or reuptake of DA. To measure the drug-induced DA release in vivo, we expressed genetically encoded fluorescent DA indicators in the brain of normal C57BL/6 J mice and used spectrally resolved fiber photometry^9^ to monitor the striatal extracellular DA level before and after the drug challenge (Fig. 2a–c). We used D1 receptor (D1R)-based DA sensor dLight1.1^10^ in this study because haloperidol, one of the candidate challenging agents, blocks the binding of DA with the D2 receptor (D2R)-based GRAB_DA_ sensors^11,12^. Although dLight1.1 and tdTomato were expressed in both cortex and the striatum, we placed the tip of the optical probe in the dorsolateral striatum to only measure the extracellular DA changes in the striatum. We measured the fluorescence change of dLight1.1 induced by intraperitoneal (i.p.) injections of 1) amphetamine, which acts on DA transporter (DAT) and releases DA^13^; 2) methylphenidate, which also acts on DAT and blocks the reuptake of DA^14^; 3) D2R antagonist haloperidol, which increases the firing rate of DANs and DA release by blocking presynaptic D2Rs (autoreceptors)^15^, and 4) the combination of methylphenidate and haloperidol, which stimulates DA release and blocks DA reuptake at the same time. Not surprisingly, we found that, compared to amphetamine (2 mg/kg), methylphenidate (10 mg/kg), and haloperidol (1 mg/kg), co-administration of methylphenidate and haloperidol produced the largest rise in the extracellular DA level (Fig. 2d, e). Thus, we selected co-administration of methylphenidate and haloperidol as the challenge protocol for DNC Test in mouse PD models.

### DNC Test can detect early-stage PD

**Fig. 3:**
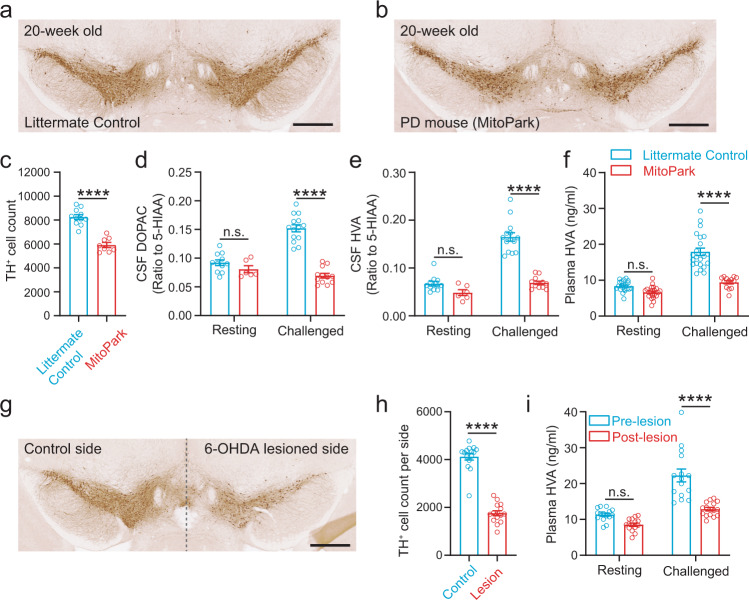
DNC Test reveals the hypodopaminergic state in early-stage PD mice with less than 30% loss of DANs. **a**, **b** DAB staining of TH^+^ cells in the ventral midbrain. Scale: 500 µm. **c** The number of TH^+^ cells counted from 11 sequential brain sections from Bregma −2.70 mm to −3.75 mm. ****, *p* < 0.0001, unpaired *t* test, *n* = 11 and 8 for 20-week-old control and MitoPark mice. **d**–**f** Levels of DA metabolites in CSF (**d**, **e**) and plasma (**f**) at the resting state and after an i.p. injection of methylphenidate (10 mg/kg) + haloperidol (1 mg/kg) in 20-week-old littermate control and MitoPark mice. n.s., *p* > 0.05; ****, *p* < 0.0001, two-way ANOVA followed by Sidak’s multiple comparisons test. *n* = 11 and 6 for control and MitoPark at resting state, *n* = 14 and 12 for control and MitoPark after the challenge in **d** and **e**; *n* = 21 and 22 for control and MitoPark at resting state, *n* = 22 and 13 for control and MitoPark after the challenge in **f**. **g**, DAB staining of TH^+^ cells in the ventral midbrain in a mouse with unilateral intrastriatal injection of 6-OHDA. Scale: 500 µm. **h**, The number of TH^+^ cells counted from 11 sequential brain sections from Bregma −2.70 mm to −3.75 mm. ****, *p* < 0.0001, paired *t* test, *n* = 15. **i**, Plasma HVA level at the resting state and after an i.p. injection of methylphenidate (10 mg/kg) + haloperidol (1 mg/kg) in C57BL/6 J mice before and after the unilateral 6-OHDA lesion. n.s., *p* > 0.05; ****, *p* < 0.0001, two-way ANOVA followed by Sidak’s multiple comparisons test. *n* = 15 for all groups of values. All data are plotted as Mean ± SEM overlaid with individual replicates.

To test the sensitivity and selectivity of the DNC Test for detecting early-stage PD, we performed DNC Test in 20-week-old MitoPark mice, a genetic PD model that shows age-dependent progressive loss of DANs^16^. At the age of 20 weeks, MitoPark mice showed 28% loss of DANs compared to age-matched littermate controls (8252 ± 204 cells per brain in 11 control mice vs. 5914 ± 213 cells per brain in 8 MitoPark mice, counted from 11 sequential and evenly spaced coronal sections from Bregma −2.70 mm to −3.75 mm, Fig. 3a–c, Supplementary Fig. 1), making them an ideal model to study early-stage PD. We compared the levels of the main DA metabolites 3,4-Dihydroxyphenylacetic acid (DOPAC) and homovanillic acid (HVA) in CSF samples and the level of HVA in plasma samples between 20-week-old MitoPark mice and their littermate controls at resting state and at 60 min after the drug challenge. Due to the extremely small volume (2–5 µl) of CSF samples that could be collected from each mouse for HPLC analysis, we used the main serotonin metabolite 5-Hydroxyindoleacetic acid (5-HIAA) in the sample as the internal control and used ratios of DOPAC/5-HIAA and HVA/5-HIAA to represent CSF DOPAC and HVA levels to minimize the errors produced by small volume handling and dilution during sample preparations (Supplementary Fig. 2). We did not compare the level of DOPAC in the plasma because, unlike HVA, DOPAC cannot cross the blood-brain barrier^17,18^. We found that there was no significant difference in the baseline values of DOPAC/5-HIAA, HVA/5-HIAA in the CSF or HVA level in the plasma between 20-week-old MitoPark mice and controls at the resting state (CSF DOPAC/5-HIAA, littermate control = 9.22 × 10^−2^ ± 4.96 × 10^−3^, MitoPark = 8.07 × 10^−2^ ± 5.96 × 10^−3^; CSF HVA/5-HIAA, littermate control = 6.71 × 10^−2^ ± 4.93 × 10^−3^, MitoPark = 4.84 × 10^−2^ ± 6.15 × 10^−3^; Plasma HVA, littermate control = 8.35 ± 0.31 ng/ml, MitoPark = 6.62 ± 0.35 ng/ml). After the drug challenge however, a large difference between PD and control with distinct data clusters was revealed (CSF DOPAC/5-HIAA: littermate control = 1.52 × 10^−1^ ± 6.21 × 10^−3^, MitoPark = 6.94 × 10^−2^ ± 4.14 × 10^−3^; CSF HVA/5-HIAA: littermate control = 1.65 × 10^−1^ ± 8.92 × 10^−3^, MitoPark = 6.90 × 10^−2^ ± 3.50 × 10^−3^; Plasma HVA: littermate control = 17.91 ± 1.04 ng/ml, MitoPark = 9.39 ± 0.46 ng/ml, Fig. 3d–f), suggesting that DNC Test can detect PD with high sensitivity when less than 30% of DANs are lost.

To further validate that DNC Test can detect a mild loss of DANs in early PD using a different PD model, we performed DNC Test in C57BL/6 J mice before and after they had received unilateral injection of 6-OHDA in the dorsal striatum. In this PD model, 57% of the DANs on the lesioned side were lost compared to the control side (4125 ± 140.1 cells on the control side vs. 1760 ± 104.6 cells on the lesioned side, counted from 11 sequential and evenly spaced coronal sections from Bregma −2.70 mm to −3.75 mm, Fig. 3g, h, Supplementary Figure 3), creating a PD model with 29% total loss of striatum-projecting DANs per animal. We compared the plasma HVA level at the resting state and after the drug challenge in these mice before and after the 6-OHDA lesion. Similar to what we observed in the 20-week-old MitoPark mice, there was no significant difference between the baseline HVA levels in the mice before and after the lesion (ng/ml, pre-lesion = 11.33 ± 0.45, postlesion = 8.55 ± 0.45), whereas the drug challenge revealed a large difference in plasma HVA levels between the pre- and post-lesion conditions (ng/ml, pre-lesion = 22.25 ± 1.81, post-lesion = 12.85 ± 0.51) (Fig. 3i).

### DNC Test can detect ultra-early stage PD

**Fig. 4:**
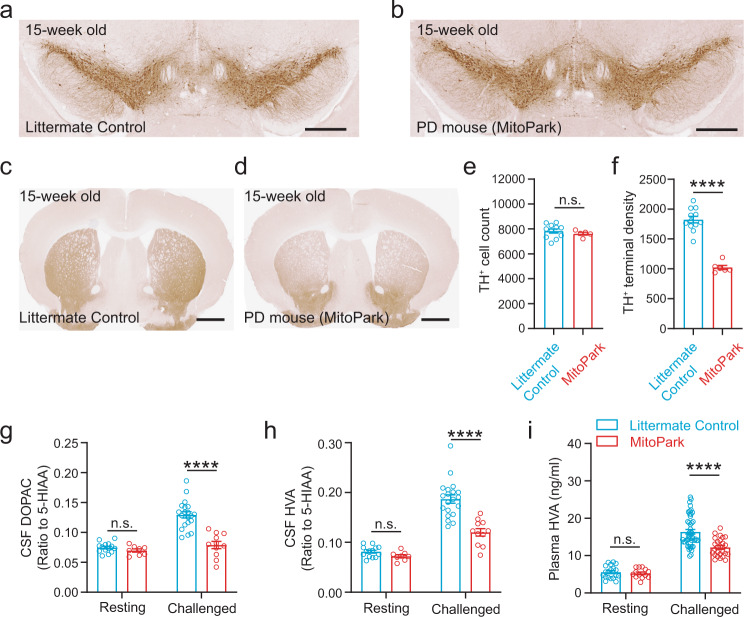
DNC Test can detect the hypodopaminergic state in MitoPark PD mice at an ultra-early stage before the loss of DANs occurs. **a**, **b**, DAB staining of TH^+^ cells in the ventral midbrain. Scale: 500 µm. **c**, **d**, DAB staining of TH^+^ terminals in the striatum. Scale: 1000 µm. **e**, The number of TH^+^ cells counted from 11 sequential brain sections from Bregma −2.70 mm to −3.75 mm. n.s., *p* > 0.05, unpaired *t* test, *n* = 11 and 5 for 15-week-old control and MitoPark mice. **f**, The TH^+^ terminal optical density from 8 sequential brain sections from Bregma 1.20 mm to −0.03 mm. ****, *p* < 0.0001, unpaired *t* test, *n* = 12 and 6 for 15-week-old control and MitoPark mice. **g**–**i**, Levels of DA metabolites in CSF (**g**, **h**) and plasma (**i**) at the resting state and after an i.p. injection of methylphenidate (10 mg/kg) + haloperidol (1 mg/kg) in 15-week-old littermate control and MitoPark mice. n.s., *p* > 0.05; ****, *p* < 0.0001, two-way ANOVA followed by Sidak’s multiple comparisons test. *n* = 14 and 8 for control and MitoPark at resting state, *n* = 20 and 11 for control and MitoPark after the challenge in **g** and **h**; *n* = 19 and 13 for control and MitoPark at resting state, *n* = 47 and 25 for control and MitoPark after the challenge in **i**. All data are plotted as Mean ± SEM overlaid with individual replicates.

Next, to test the feasibility of using the DNC Test to detect the ultra-early stage PD when the loss of DANs has not occurred, we performed DNC Test in 15-week-old MitoPark and littermate control mice. At the age of 15 weeks, MitoPark mice have not developed significant loss of DANs compared to age-matched littermate controls (7830 ± 168 cells per brain in 11 control mice vs. 7618 ± 115 cells per brain in five MitoPark mice, counted from 11 sequential and evenly spaced coronal sections from Bregma −2.70 mm to −3.75 mm, Fig. 4a, b, e, Supplementary Fig. 4). The major pathological change at this stage was 44% loss of TH^+^ axonal terminals in the dorsal striatum (TH^+^ terminal optical density: 1823 ± 186 in 12 control vs. 1023 ± 89 in six MitoPark mice, averaged from eight sequential brain sections from Bregma 1.20 mm to −0.03 mm, Fig. 4c, d, f, Supplementary Fig. 5), making them an ideal model for studying the ultra-early stage of PD. We compared the ratios of DOPAC/5-HIAA and HVA/5-HIAA in CSF samples and the level of HVA in plasma samples between 15-week-old MitoPark mice and their littermate controls at resting state and at 60 min after the drug challenge. We found that there was no significant difference in the baseline values of DOPAC/5-HIAA, HVA/5-HIAA in the CSF or HVA level in the plasma between MitoPark mice and controls at the resting state (CSF DOPAC/5-HIAA, littermate control = 7.46 × 10^−2^ ± 2.24 × 10^−3^, MitoPark = 6.97 × 10^−2^ ± 2.75 × 10^−3^; CSF HVA/5-HIAA, littermate control = 8.13 × 10^−2^ ± 2.92 × 10^−3^, MitoPark = 7.22 × 10^−2^ ± 3.21 × 10^−3^; Plasma HVA ng/ml, littermate control = 5.58 ± 0.39, MitoPark = 5.28 ± 0.36). After the drug challenge however, a significant difference in these measurements was revealed (CSF DOPAC/5-HIAA: littermate control = 1.29 × 10^−1^ ± 5.27 × 10^−3^, MitoPark = 7.88 × 10^−2^ ± 6.03 × 10^−3^; CSF HVA/5-HIAA: littermate control = 1.87 × 10^−1^ ± 8.37 × 10^−3^, MitoPark = 1.20 × 10^−1^ ± 7.68 × 10^−3^; Plasma HVA ng/ml: littermate control = 16.37 ± 0.60, MitoPark = 12.21 ± 0.49, Fig. 4g–i).

### Effects of DNC Test on locomotor activity

**Fig. 5:**
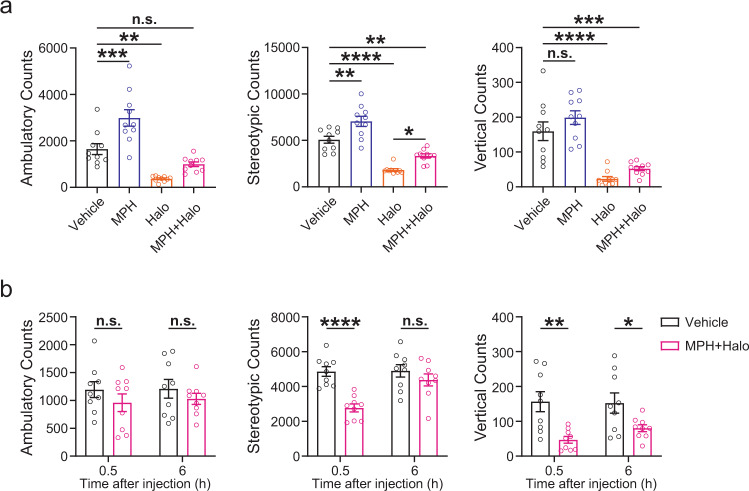
DNC Test causes mild and reversible inhibition on voluntary movement in normal C57BL/6 J mice. **a**. Ambulatory (*left*), stereotypic (*middle*), and vertical (*right*) counts in an open-field test 30 min after an i.p. injection of the vehicle (saline), 10 mg/kg methylphenidate, 1 mg/kg haloperidol, or co-administration of methylphenidate and haloperidol (DNC Test agents). n.s., *p* > 0.05; *, *p* < 0.05; **, *p* < 0.01; ***, *p* < 0.001; ****, *p* < 0.0001, one-way ANOVA followed by Dunnett’s multiple comparisons test. *n* = 10 C57BL/6 J mice for each group. All data are plotted as Mean ±SEM overlaid with individual replicates. **b**. Ambulatory (*left*), stereotypic (*middle*), and vertical (*right*) counts in an open-field test performed at 0.5 h and 6 h after an i.p. injection of vehicle (DMSO) or DNC Test agents methylphenidate (10 mg/kg) and haloperidol (1 mg/kg). n.s., *p* > 0.05; *, *p* < 0.05; **, *p* < 0.01; ****, *p* < 0.0001, two-way ANOVA followed by Sidak’s multiple comparisons test. *n* = 10 C57BL/6 J mice for each group. All data are plotted as Mean ±SEM overlaid with individual replicates.

Finally, since both methylphenidate and haloperidol are dopaminergic drugs that are known to affect locomotor activity, we examined the potential side effects of the DNC Test on locomotor activity by performing open-field tests in normal C57BL/6 J mice after the administration of vehicle (normal saline), methylphenidate (10 mg/kg), haloperidol (1 mg/kg), and the DNC Test agents methylphenidate (10 mg/kg) + haloperidol (1 mg/kg). We found that co-administration of methylphenidate alleviated the inhibitory effect of haloperidol on locomotor activity (Fig. 5a), and that the locomotor activity mostly recovered to normal levels within 6 h after the drug injection (Fig. 5b), suggesting that DNC Test-induced inhibition on movement was mild and reversible.

## Discussion

In the fiber photometry experiment where we used dLight1.1 to measure striatal DA release, dLight1.1 and tdTomato were expressed in both cortex and the striatum (Fig. 2b). This was because the experiment was carried out in wild-type mice with non-Cre-dependent viral vectors. Cortical expression of dLight1.1 does not affect the interpretation of the results because the tip of the recording optical fiber was placed inside the striatum. The small (0.22) numerical aperture (N.A.) of the fiber we used and the limited (~500 µm) detection depth in brain tissue^19^ would limit the detection of fluorescence signals within the striatum.

In this study, the CSF and plasma samples were collected under anesthesia, which raises the concern that the anesthesia could affect the circulating levels of the metabolites measured. For CSF collections, the surgical procedure typically took less than 5 min to complete. It has been shown previously that isoflurane anesthesia does not cause major changes in the extracellular concentrations of DOPAC or HVA within the first 20 min of anesthesia^20^. For blood sample collections, mice were only briefly anesthetized in the isoflurane induction chamber for less than one minute before the blood draw. It was very unlikely that this brief anesthesia would significantly affect the levels of DA metabolites in the blood samples. Furthermore, CSF and blood sample collections in humans do not require general anesthesia. Thus, anesthesia won’t be a confounding factor for DNC Test in humans.

Our DNC Test results in PD models suggest that at the early stage of PD when the DAN loss is less than 30%, the baseline levels of DA metabolites in the CSF and plasma samples from PD patients are not distinguishable from the healthy controls at the resting state. However, a drug challenge that stimulates DA release can help reveal the differences. Our results show that using CSF samples, DNC Test can achieve 100% sensitivity and specificity in detecting the hypodopaminergic state in 20-week-old MitoPark mice with 28% loss of DANs, and 91% sensitivity and 80–85% specificity in 15-week-old MitoPark mice with no detectable loss of DANs (Table 1). Using plasma samples, DNC Test can achieve 100% sensitivity and 82% specificity in 20-week-old MitoPark mice, and 76% sensitivity and 60% specificity in 15-week-old MitoPark mice (Table 1). Plasma samples are easy to collect, making them an ideal choice for general screening. Using CSF samples, DNC Test provides superior separation of the data clusters between the PD and control groups (Fig. 3d, e, Fig. 4g, h), making CSF samples a better option for confirmatory tests during the follow-up examination in suspicious cases. Importantly, when we replot the data separating male and female mice, DNC Test is equally effective in detecting PD in both genders (Supplementary Fig. 6), making it potentially suitable for diagnosing early PD in both male and female patients in humans.

**Table 1:** Sensitivity and specificity of DNC Test for detecting early and ultra-early stage PD in MitoPark mice.

PD stage	DNC Test parameter	Threshold for positive diagnosis (Mean_control_ + Mean_PD_)/2	Sensitivity (true positive rate)	Specificity (true negative rate)
Early (~30% loss of DANs)	CSF DOPAC/5-HIAA	0.1107	100% (12/12)	100% (14/14)
	CSF HVA/5-HIAA	0.1169	100% (12/12)	100% (14/14)
	Plasma HVA (ng/ml)	13.65	100% (13/13)	82% (18/22)
Ultra-early (no loss of DANs)	CSF DOPAC/5-HIAA	0.1040	91% (10/11)	85% (17/20)
	CSF HVA/5-HIAA	0.1535	91% (10/11)	80% (16/20)
	Plasma HVA (ng/ml)	14.29	76% (19/25)	60% (28/47)

To be eligible as a screening or diagnostic test in clinical settings, it is crucial that the test must be safe and without severe adverse side effects. Both methylphenidate and haloperidol are FDA-approved drugs and have been in clinical use for decades. However, a major concern and potential limitation of using haloperidol in DNC Test is that haloperidol may cause extrapyramidal symptoms in elderly adults due to its blocking effect on postsynaptic D2Rs^21^. Our open-field results in healthy C57BL/6 J mice show that co-administration of methylphenidate alleviates haloperidol-induced inhibition on locomotor activity, suggesting that the DNC Test has a lower risk of causing severe extrapyramidal symptoms compared to using haloperidol alone (Fig. 5). Co-administration with anticholinergic drugs^22^ and limiting the testing age to 65 or younger^23^ may also help reduce the risks of severe side effects. Furthermore, the findings in this preclinical study mainly serve as a proof of concept to demonstrate that DNC Test can detect the loss of DANs in early PD. There are many other candidate drugs that act on presynaptic D2Rs and can induce robust DA release without causing extrapyramidal side effects^24^. Their effectiveness and safety in DNC Test can be explored in clinical trials in order to find the best challenging agents for use in humans.

In summary, we proposed a new laboratory test for diagnosing early PD and tested the hypothesis that challenging DANs pharmacologically can reveal the loss of DANs at the early stage of PD. Using two mouse PD models with mild loss of DANs, we show that DNC Test can reliably detect the differences between PD and control mice using CSF and plasma samples.

## Methods

### Animals

All animal protocols were approved by the US National Institute of Environmental Health Sciences Animal Care and Use Committee. Adult male and female C57BL/6 J mice were acquired from the Jackson Laboratory. MitoPark mice (DAT^IRES*cre*/+^;Tfam^flox/flox^) were bred in house by crossing DAT^IRES*cre*/+^ mice^25^ (006660, Jackson Laboratory) with Tfam^flox/flox^ (026123, Jackson Laboratory). Tfam^flox/flox^ and DAT^IRES*cre*/+^;Tfam^flox/+^ mice were used as breeders to generate MitoPark mice. Tfam^flox/flox^, Tfam^flox/+^ and DAT^IRES*cre*/+^;Tfam^flox/+^ mice were used as littermate controls.

### Viral vectors

pAAV-CAG-dLight1.1 was a gift from Lin Tian (Addgene plasmid # 111067; http://n2t.net/addgene:111067; RRID:Addgene_111067)^10^. pAAV-hSyn1(S)-tdTomato-WPRE was a gift from Hongkui Zeng (Addgene plasmid # 51506; http://n2t.net/addgene:51506; RRID:Addgene_51506)^26^. All viral vectors were recovered in house by the NIEHS Viral Vector Core and had titers of 1.3 × 10^13^ to 2 × 10^14^ genome copies per ml and packaged with AAV9 capsid.

### Viral expression of dLight1.1 and tdTomato control

Viral vectors were micro-injected into the left and right dorsal striatum by standard stereotaxic procedures with animals under isoflurane anesthesia^27^. The coordinates used for targeting the dorsal striatum were AP + 0.50 mm, ML ± 2.30 mm from Bregma; and DV −2.75 mm from the brain surface. A total volume of 1.0 µl of AAV vectors per site was injected at the rate of 0.1 µl per min through a Hamilton Neuros syringe with a 30-gauge needle. The needle was left in place for 10 more minutes before the withdrawal. The volume ratio of viral vectors in the mixtures for injections was 30:1 for dLight1.1 and tdTomato.

### Optical fiber probe implantation for in vivo fiber photometry recordings in normal C57BL/6 J mice

Two weeks after the AAV injection, normal C57BL/6 J mice underwent the second stereotaxic surgery to receive fiber probe implantation. Two bur holes were drilled though the skull to target the dorsal striatum bilaterally (AP + 0.50 mm, ML ± 2.40 mm from Bregma) using a #1/2 (0.027” Diameter) drill bit. Another pair of bur holes for anchoring screws were drilled bilaterally above the parietal lobes. After the anchoring screws were in place, a lab-made optical fiber probe, fabricated using a multimode fiber (Thorlabs, FG105UCA, NA: 0.22, core diameter: 105 µm, total fiber diameter with cladding: 125 µm, fiber length: flat-cleaved to 2.5 mm after probe assembling and before use) and a ceramic ferrule disassembled from commercial LC connectors (Precision Fiber Products, Inc. SKU: MM-CON2010, ferrule outer diameter: 1.25, bore diameter: 127 µm) was slowly lowered onto the surface of the cortex through the burr hole, then further lowered toward the dorsal striatum at approximately 200 µm per step until the spectra of dLight1.1 and tdTomato were detected by the spectrometer. The probe was then lowered at 50 µm per step until the fluorescence intensity reached a plateau. The final tip location was approximately 1.8–2.2 mm below the brain surface. The probe was then fixed in place with a generous amount of dental acrylic (Jet, Lang Dental Mfg. Co.). The animals were allowed to recover for 2 weeks before experiments proceeded.

### Spectrally resolved fiber photometry

A blue laser (473 nm) was used as the light source to excite both dLight1.1 and tdTomato. Emitted photons were collected by a spectrometer as described previously^9^. The in vivo recordings in awake behaving mice were carried out in an open-top mouse operant chamber (21.6 × 17.8 × 12.7 cm, Med-Associates) housed in a sound-attenuating box. Fluorescence spectra were acquired using 19 ms integration time and were triggered by 25 Hz TTL pulses sent from a digital output module (DIG-726TTL, Med-Associates) on a customized mouse operant conditioning package from Med-Associates. Spectral linear unmixing was carried out using a customized program written in R. The customized spectral linear unmixing algorithm script written in R is available at https://www.niehs.nih.gov/research/atniehs/labs/ln/pi/iv/tools/index.cfm. A digital video camera (Grasshopper3 GS3-U3-23S6M-C, FLIR Integrated Imaging Solutions), frame by frame triggered by the same TTL pulses triggering the spectrometer, was used to record the animal’s behavior. The output power of the 473 nm laser measured at the end of the patch cable was set at 50 µW. Under these conditions, we found that the dLight1.1 fluorescence showed significant fading during the recordings. To correct for the fluorescence fading, we first applied linear regression to fit the first 10 min of the unmixed coefficients plotted over time, and then used the fitted curve as the theoretical baseline (F_0_) to calculate ΔF/F_0_%.

### CSF and plasma sample collections during the DNC Test

CSF and blood samples were collected separately from different mice 1 hr after the i.p. injection of the mixture of methylphenidate (10 mg/kg) and haloperidol (1 mg/kg).

CSF was collected through cisterna magna puncture^28^. The mice were anesthetized by 5% isoflurane in the induction chamber and then transferred to a stereotaxic frame where the anesthesia was maintained with 1–2% isoflurane. The mice were placed on the stereotaxic frame in the way that the head formed a ~135 ° angle with the body. A sagittal incision was made above the occiput. The subcutaneous tissue and muscles were separated with forceps. A glass capillary (1B150-4, WPI) pulled by a puller (PC-10, NARISHIGE Group) with the tip trimmed with scissors was used to penetrate into the cisterna manga through the dura mater to collect CSF. The collected CSF was injected into an Eppendorf tube with a 0.3 ml insulin syringe (324702, BD Biosciences). After the CSF collection, the muscles were re-aligned, and the skin was sutured. The mice were either perfused or sacrificed after CSF collection. The CSF samples were either put on ice for immediate HPLC analysis or stored at −80 °C.

Blood was collected through submandibular bleeding^29^. Mice were anesthetized by 5% isoflurane in the induction chamber. The cheek was shaved, and a 22-gauge needle was used to puncture the submandibular vein. Drops of blood (0.2~0.5 ml) were collected with heparin pre-coated tubes (NC9016222, Fisher Scientific). The penetration point was pressed with cotton swaps to stop the bleeding after the blood collection. The mice were housed on heat pad until ambulatory. Blood samples were centrifuged at 2000 g × 5 min at 4 ^o^ C. The clear supernatants (plasma) were placed on ice for immediate HPLC analysis or stored at −80 °C.

### Sample preparation and HPLC analysis of DA metabolites

Sixty to Sixty-two percentage perchloric acid (9.5 M PCA, 33263, Thermo Fisher Scientific) was diluted with water to 1 M and stored at room temperature. CSF (2–5 µl) samples were mixed with 10 µl 1 M PCA. 20 µl plasma samples were mixed with 50 µl 1 M PCA. The mixtures were vortexed immediately and centrifuged at 18,000 g × 15 min at 4 ^o^ C. The clear supernatant was collected and filtered with microcentrifuge filter (18,000 g × 5 min at 4 ^o^ C, 8169, Costar) before the HPLC assay.

Purchased reference standards for DA (43658-50MG, Sigma-Aldrich), DOPAC (11569-25MG, Sigma-Aldrich), HVA (H1252-100MG, Sigma-Aldrich), and 5-HIAA (55697-10MG, Sigma-Aldrich) were dissolved in methanol (A456-4, Fisher Chemical) to make 1 mg/ml stock and stored at −80 ^o^ C. The stock solutions were further diluted with 1 M PCA to make final DA (100 ng/ml), DOPAC (10 ng/ml), HVA (10 ng/ml), and 5-HIAA (10 ng/ml) solutions for use as reference standards.

The Dionex UltiMate 3000 UHPLC system consisted of an ISO-3100BM pump, a WPS-3000 autosampler, an ECD-3000RS electrochemical detector with 6020RS omni coulometric cell and 6041RS ultra amperometric cell, connected to a computer equipped with Chromeleon 7 chromatography software. The coulometric cell was set to −100 mV, and the amperometric cell was set to 300 mV. The mobile phase buffer (Thermo Scientific MDTM 70-1332, NC9777698, Fisher Scientific) was pumped at a flow rate of 0.75 mL/min through an Acclaim™ 120 C18 column (3.0 × 100 mm, Thermo Fisher Scientific). The injection volume was 5 µl for CSF and the mixture of reference standards and was 40 µl for plasma samples. The retention time of the standards, CSF samples, and plasma samples were 5, 10, and 13 min, respectively.

The area under the curve in the chromatogram was used to calculate the concentrations of the components detected by Chromeleon 7. The ratios of DOPAC/5-HIAA and HVA/5-HIAA were used to represent the relative concentrations of DOPAC and HVA in the CSF. To calculate the absolute concentration of HVA in plasma samples, a standard curve was generated by running HPLC on samples made of 20 µl HVA reference standard of different concentrations (0.5, 1, 5, 10, 20, 30, 40, 50 ng/ml) mixed with 50 µl 1 M PCA, with 40 µl injection volume.

### DNC Test in the unilateral 6-OHDA lesion PD model

Blood samples were collected from male and female C57BL/6 J mice at the resting state and after the drug challenge (an i.p. injection of 10 mg/kg methylphenidate and 1 mg/kg haloperidol) at the age of 20 and 25 weeks before 6-OHDA lesion. The samples collected at the resting and challenged states were counterbalanced between these two dates to avoid the potential impact of age-related change in plasma HVA level over time. At the age of 30 weeks, the mice received an i.p. injection of 25 mg/kg desipramine hydrochloride and 5 mg/kg pargyline hydrochloride dissolved in 0.9% saline 30 min before the 6-OHDA injection. The neurotoxin 6-OHDA (5 µg in 2 µl total volume, 0.2 ml/min) was injected unilaterally (counterbalanced between left and right) into the striatum. The stereotaxic coordinates for the injection site were AP + 0.70 mm, ML ± 1.50 mm from Bregma; and DV −3.00 mm from the brain surface. Blood samples were collected again at 4 and 9 weeks after the surgery (counterbalanced between the resting and challenged states).

### Open-field test in normal C57BL/6 J mice

The open-field test was carried out in a seamless open-field chamber (27.31 × 27.31 × 20.32 cm, ENV-510, Med-Associates) housed in a sound-attenuating box (ENV-018MD-EMS-27, Med-Associates). C57BL/6 J mice were placed in the chamber 30 min or 6 h after injection for a 30-min measurement. Movements were tracked by three 16-beam IR arrays located on the X and Y axes for positional tracking and on the Z axis for rearing detection. Ambulatory counts, stereotypic counts, and vertical counts were used to represent the voluntary movement of mice.

### Immunohistochemistry

Mice were transcardially perfused with phosphate-buffered saline (PBS) followed by 4% paraformaldehyde (PFA). Brains were post-fixed in 4% PFA for 24 h, and then transferred to 30% sucrose in PBS for storage at 4 ^o^C. Coronal slices were sectioned on a microtome (KS34, Thermo Fisher Scientific) at the thickness of 35 µm.

For DAB staining, sections were blocked with 10% normal goat serum (Vector Labs) and 0.2% Triton X-100 (Sigma) for 1 hr at room temperature, followed by incubation at 4 ^o^C with a primary antibody against tyrosine hydroxylase (TH) (1:4000, ab152, Millipore/Chemicon) overnight. Sections were then incubated with a biotinylated secondary antibody (1:200, BA-1000, Vector Laboratories) for 1 hr, followed by incubation in avidin-biotin-peroxidase complex (PK6100, Vector Laboratories) for 1 hr, and immunoreactivity was detected with 3,3’-diaminobenzidine (DAB, D5905, Sigma-Aldrich). The tissue was then dehydrated and cleared with ethanol and xylenes prior to coverslipping. Tissue was imaged at the NIEHS Image Analysis Group using an Aperio AT2 slide scanner (Leica Biosystems).

For immunofluorescence staining, sections were blocked with 10% normal goat serum (Vector Labs) and 0.2% Triton X-100 (Sigma) for 1 hr at room temperature, followed by incubation at 4^o^ C overnight with primary antibodies against TH (1:4000, ab152, Millipore/Chemicon) and anti-GFP (1:1000, ab13970, Abcam) for detecting dLight1.1. After washing out excessive primary antibodies, the slices were incubated in the secondary antibodies for 2 hr at room temperature. The secondary antibodies used were Alexa Fluor 488 conjugated goat anti-chicken (1:500, A-11039, Invitrogen), and goat antirabbit Alexa Fluor 647 (1:1000, A-21245, Invitrogen). After thoroughly washing, the slices were mounted on slides and imaged on a Zeiss Axiocam MR monochrome camera installed on Axio observer Z1 fluorescent microscope with 20x objective (numerical aperture 0.8). The images were acquired and processed using Zen 2012 Blue software (Carl Zeiss).

### TH^+^ cell counting

RGB color images (11 sections/animal) were analyzed using ImageJ (1.8.0_60) with the experimenter blind to the genotypes or treatment conditions. The image was first split into its RGB components, and then the green channel was used for analysis since it gave the highest contrast of the DAB staining. The image for the green channel was inverted and then an eliminate maxima filter (size 7) from the Fast Filters plugin, Gaussian blur filter (size 7), and background subtraction (size 60) were used to identify the DAB stained cell bodies. Next, a mask was created by using the default automatic threshold where we performed a watershed algorithm for segmentation and then counted each particle over 100 pixels with the Analyze Particles feature. Results of the counted features were then confirmed visually by overlaying the ROI for each counted object back to the original DAB stained image to ensure accuracy.

### Quantification of TH^+^ terminal density

RGB color images (8 sections/animal) were analyzed using ImageJ (1.8.0_60) with the experimenter blind to the genotypes. Each image was split into its RGB components from which an intensity image for DAB was extracted, converted to grayscale, and inverted. The region of interest (ROI) was outlined and threshold values for pixel intensity were set from 45 to 255. Gray pixel values of images were calibrated to a density standard from an optical density grayscale. The mean gray value of the dorsal striatum was used to represent the dopamine terminal intensity for each section.

### Sensitivity and specificity for DNC Test

The threshold for a positive diagnosis was defined as the mean of two means of the challenged groups: Threshold = (Mean_challenged___control_ + Mean_challenged_PD_)/2. To calculate the sensitivity and specificity for DNC Test, we defined N_True+_ as the number of MitoPark mice that were correctly diagnosed, that is, the number of MitoPark mice with measured DNC Test values smaller than the defined threshold; N_Total_PD_ as the total number of MitoPark mice that were tested; N_True-_ as the number of littermate control mice that were correctly diagnosed, that is, the number of littermate control mice with measured DNC Test values larger than the defined threshold; N_Total_control_ as the total number of littermate control mice tested.

$${{{{{\mathrm{Sensitivity}}}}}} = {{{{{\mathrm{N}}}}}}_{{{{{{\mathrm{True}}}}}} + }{{{{{\mathrm{/ N}}}}}}_{{{{{{\mathrm{Total}}}}}}\_{{{{{\mathrm{PD}}}}}}} \times {{{{{\mathrm{ }}}}}}100\% ;{{{{{\mathrm{ Specificity}}}}}} = {{{{{\mathrm{N}}}}}}_{{{{{{\mathrm{True}}}}}} - }{{{{{\mathrm{/ N}}}}}}_{{{{{{\mathrm{Total}}}}}}\_{{{{{\mathrm{control}}}}}}} \times {{{{{\mathrm{ }}}}}}100\%$$

### Statistical analysis

One-way ANOVA, two-way ANOVA followed by multiple comparisons, unpaired and paired *t* tests were carried out using GraphPad Prism 8 (GraphPad Software). Results of the statistical analyses, including Mean ± SEM, sample sizes and *P* values are indicated in either the text or figure legends.

### Reporting Summary

Further information on research design is available in the Nature Research Reporting Summary linked to this article.

## Supplementary information

Supplementary Information

Reporting Summary

## Data Availability

All data needed to evaluate the conclusions in the paper are present in the paper and/or the Supplementary Materials. The raw and analyzed datasets generated during the study are available for research purposes from the corresponding author on reasonable request. Additional data related to this paper may be requested from the authors.
