# Supplementary material for: Dopamine Neuron Challenge Test for early detection of Parkinson’s disease
Source: NPJ Parkinsons Dis. 2021 Dec 16;7:116. doi: 10.1038/s41531-021-00261-z (PMC8677804; doi:10.1038/s41531-021-00261-z)
Supplement: Supplementary file 1 — Supplementary Information [file 41531_2021_261_MOESM1_ESM.pdf]

Supplemental Information

Dopamine Neuron Challenge Test for early detection of Parkinson's disease

Jingheng Zhou, Jicheng Li, Amy B. Papaneri, Nicholas P. Kobzar, Guohong Cui

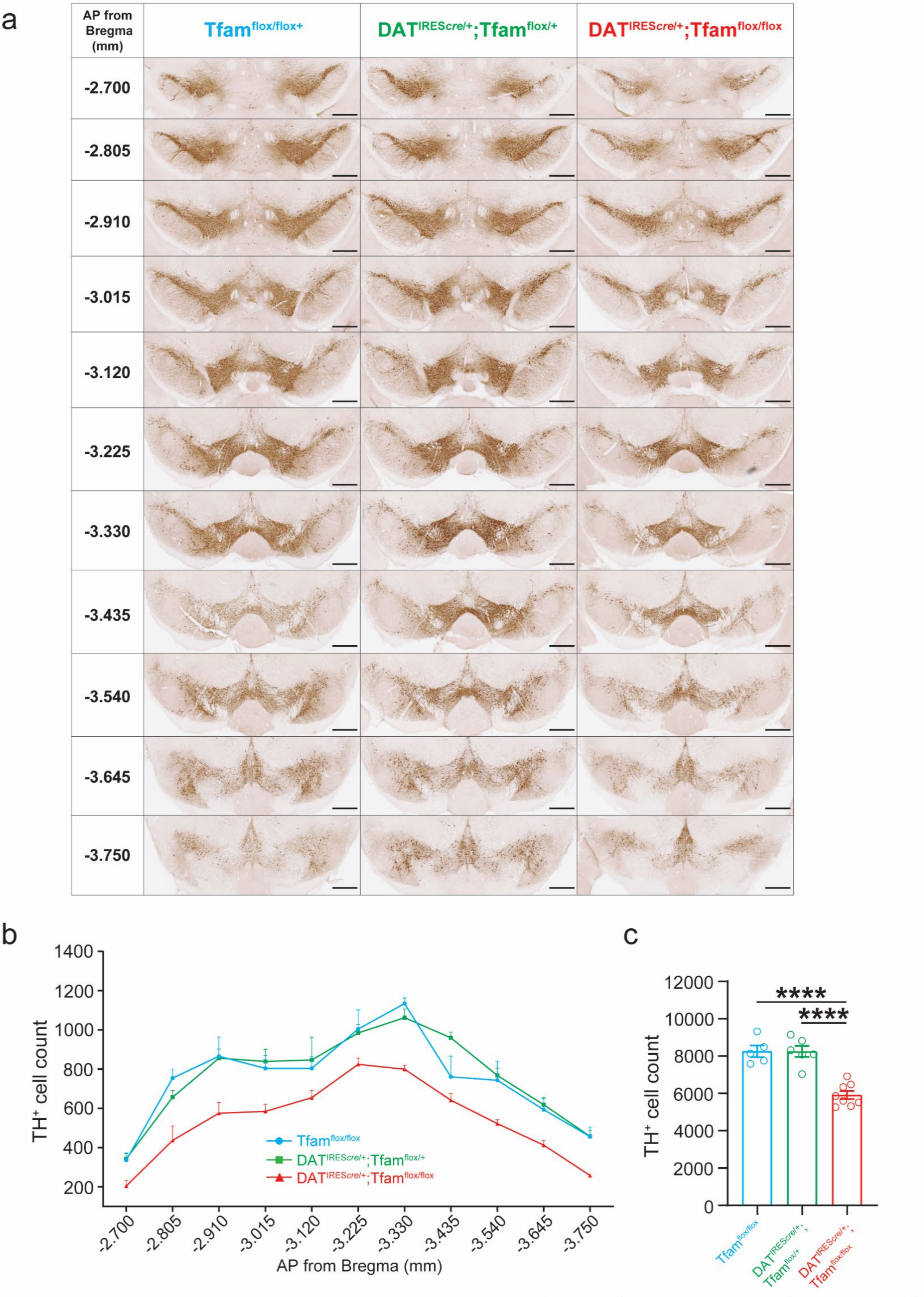

Supplementary Figure 1

Supplementary Figure 1 | Images of DAB staining for TH and results of DA neuron counting from 20-week-old littermate control and MitoPark mice.

a. Representative images of DAB staining for TH from a littermate control $Tfam^{flox/flox}$ mouse, a littermate control $DAT^{IREScre/+}$;$Tfam^{flox/+}$ mouse, and a MitoPark $DAT^{IREScre/+}$;$Tfam^{flox/flox}$ mouse. Coronal slices were sectioned at a thickness of 35 µm and selected one in every 3 sections for staining. Scale: 500 µm.

b. Cell counting results (Mean ± SEM) plotted from anterior to posterior sections.

c. Summary of the TH^+ cell counting from littermate controls with different genotypes and MitoPark mice. ****, $p < 0.0001$, one-way ANOVA followed by Dunnett's multiple comparisons test. n = 5 for $Tfam^{flox/flox}$, n = 6 for $DAT^{IREScre/+}$;$Tfam^{flox/+}$, n = 8 for MitoPark $DAT^{IREScre/+}$;$Tfam^{flox/flox}$. All data are plotted as Mean ± SEM overlaid with individual replicates.

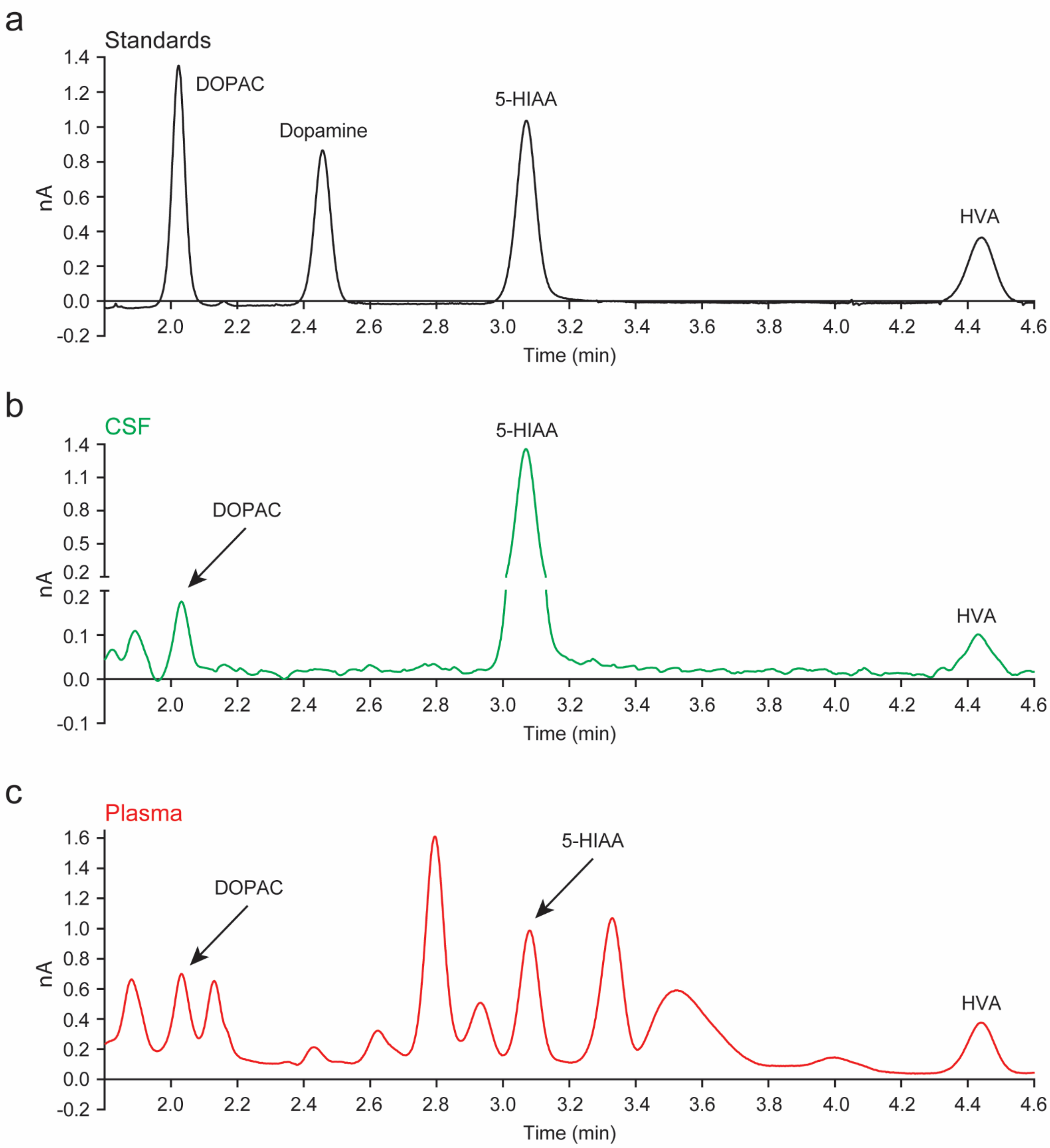

Supplementary Figure 2

Supplementary Figure 2 | Representative HPLC chromatograms of DA, DOPAC, HVA and 5-HIAA measured from standards, CSF and plasma samples.

a. A representative chromatogram of external standards of dopamine (100 ng/ml), DOPAC (10 ng/ml), HVA (10 ng/ml) and 5-HIAA (10 ng/ml) dissolved in 1 M PCA solution.

b. A representative chromatogram of a sample containing 5 µl CSF mixed with 10 µl 1 M PCA.

c. A representative chromatogram of a sample containing 20 µl plasma mixed with 50 µl 1 M PCA.
The injection volume for CSF samples and the external standards was 5 µl, for plasma was 40 µl.

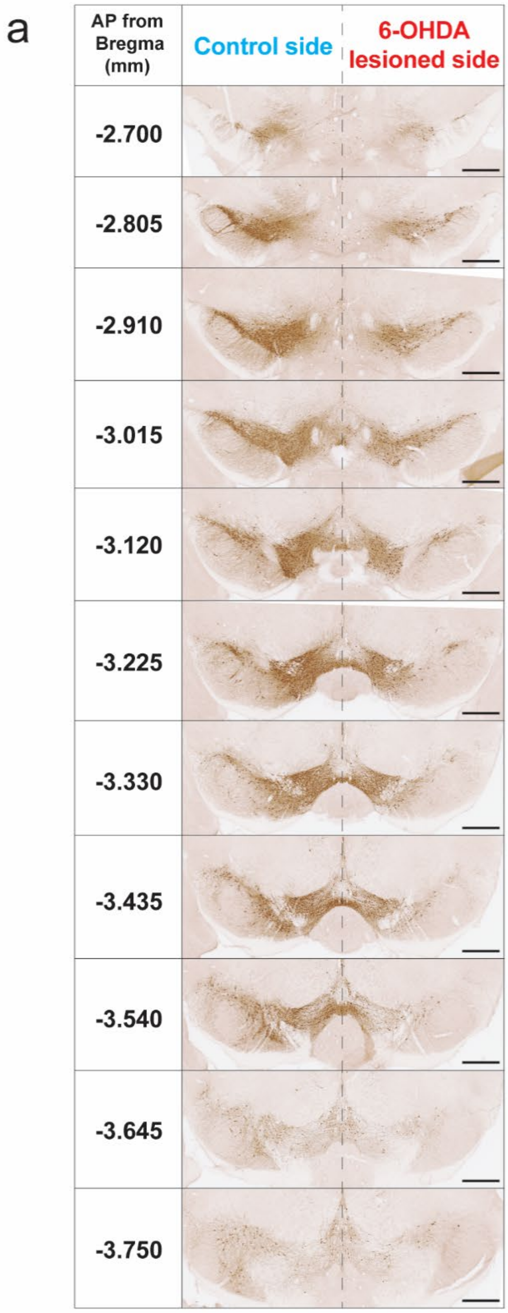

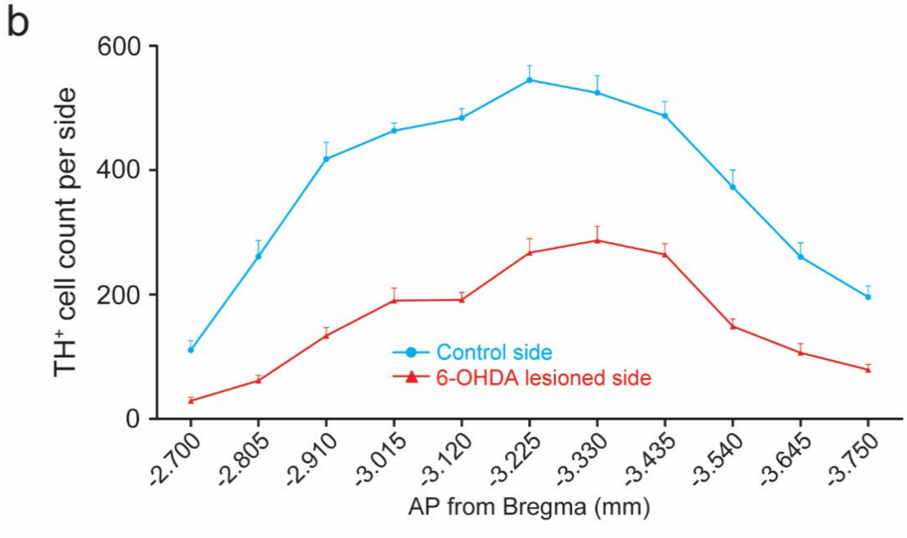

Supplementary Figure 3

Supplementary Figure 3 | Images of DAB staining for TH and results of DA neuron counting from unilateral 6-OHDA lesioned mice.

a. Representative images of DAB staining for TH from a mouse that received a unilateral 6-OHDA injection in the dorsal striatum. Coronal slices were sectioned at a thickness of 35 μm and selected one in every three sections for staining. Scale: 500 μm.

b. Cell counting results (Mean ± SEM) plotted from anterior to posterior sections.

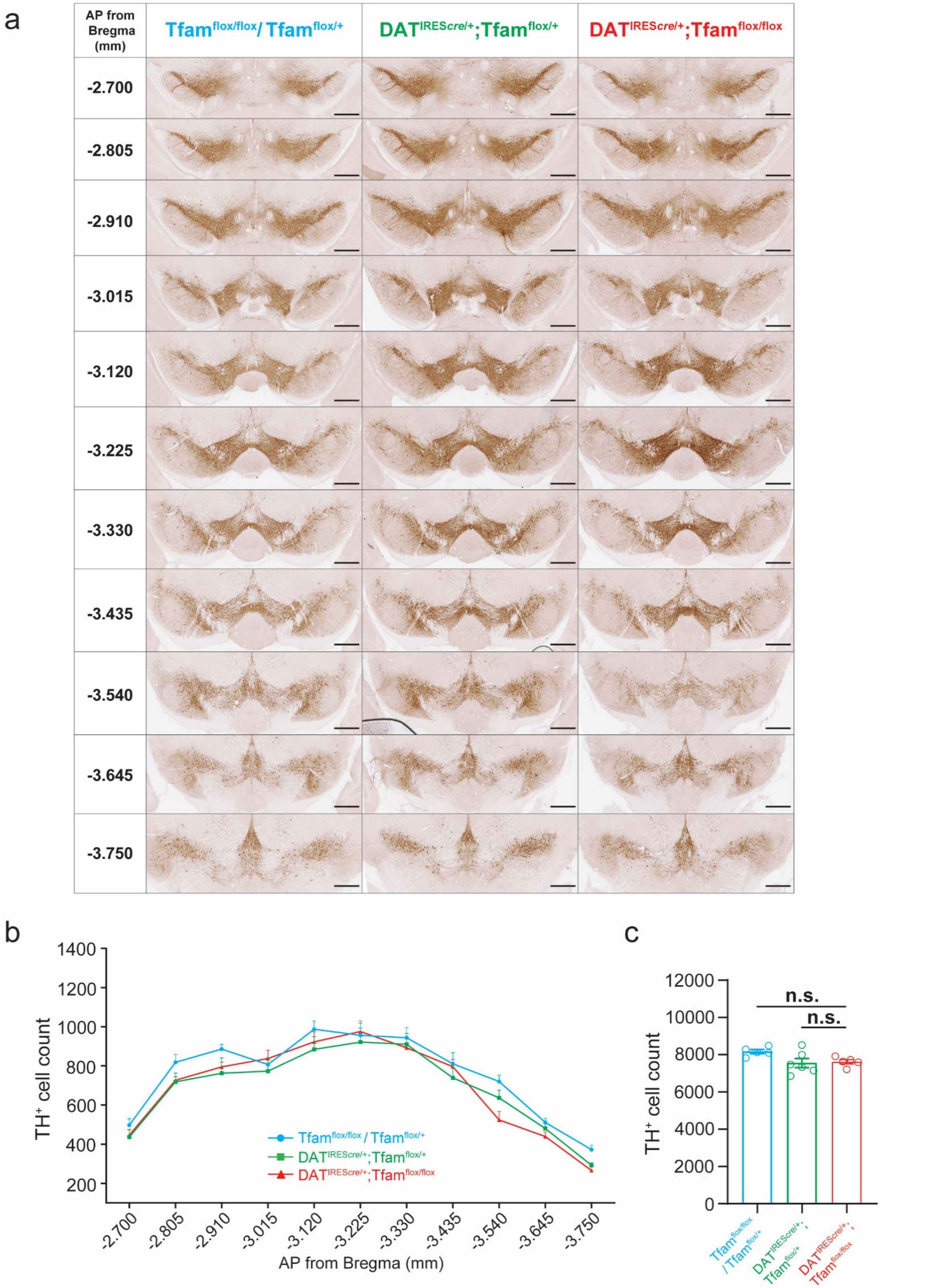

Supplementary Figure 4

Supplementary Figure 4 | Images of DAB staining for TH and results of DA neuron counting from 15-week-old littermate control and MitoPark mice.

a. Representative images of DAB staining for TH from a littermate control $Tfam^{flox/flox}$ mouse, a littermate control $DAT^{IREScre/+}$;$Tfam^{flox/+}$ mouse, and a MitoPark $DAT^{IREScre/+}$;$Tfam^{flox/flox}$ mouse. Coronal slices were sectioned at a thickness of 35 µm and selected one in every 3 sections for staining. Scale: 500 µm.

b. Cell counting results (Mean ± SEM) plotted from anterior to posterior sections.

c. Summary of the TH^+ cell counting from littermate controls with different genotypes and MitoPark mice. n.s., $p > 0.05$, one-way ANOVA followed by Dunnett's multiple comparisons test. n = 6 for $Tfam^{flox/flox}$, n = 5 for $DAT^{IREScre/+}$;$Tfam^{flox/+}$, n = 5 for MitoPark $DAT^{IREScre/+}$;$Tfam^{flox/flox}$. All data are plotted as Mean ± SEM overlaid with individual replicates.

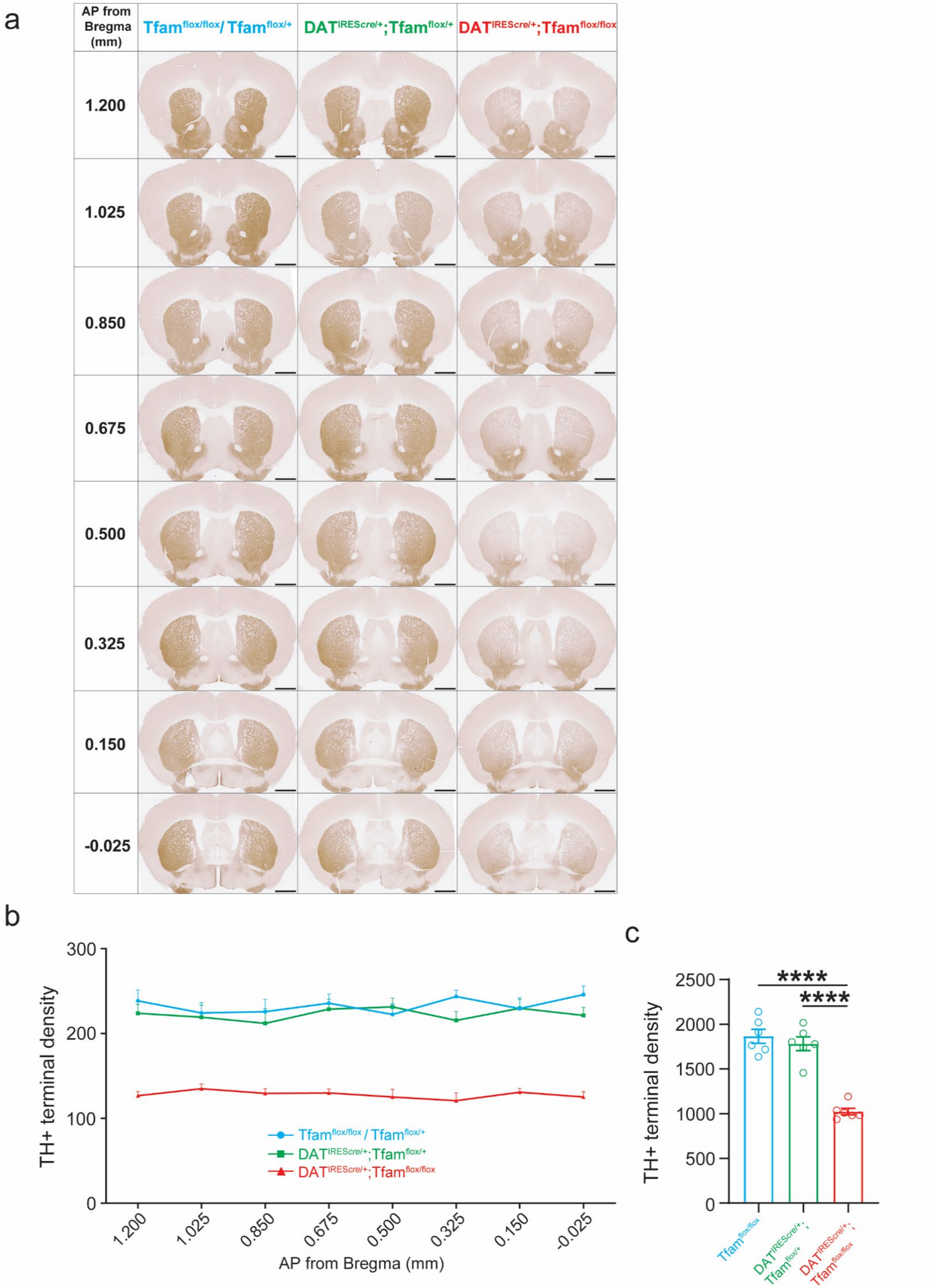

Supplementary Figure 5

Supplementary Figure 5 | Images of DAB staining for TH and results of DA neuron terminal density from 15-week-old littermate control and MitoPark mice.

a. Representative images of DAB staining for TH from a littermate control $Tfam^{flox/flox}$ mouse, a littermate control $DAT^{IREScre/+}$;$Tfam^{flox/+}$ mouse, and a MitoPark $DAT^{IREScre/+}$;$Tfam^{flox/flox}$ mouse. Coronal slices were sectioned at a thickness of 35 µm and selected one in every 5 sections for staining. Scale: 1000 µm.

b. Dopaminergic terminal density results (Mean ± SEM) plotted from anterior to posterior sections.

c. Summary of the TH^+ terminal density from littermate controls with different genotypes and MitoPark mice. ****, $p < 0.0001$, one-way ANOVA followed by Dunnett's multiple comparisons test. n = 6 for $Tfam^{flox/flox}$, n = 6 for $DAT^{IREScre/+}$;$Tfam^{flox/+}$, n = 6 for MitoPark $DAT^{IREScre/+}$;$Tfam^{flox/flox}$. All data are plotted as Mean ± SEM overlaid with individual replicates.

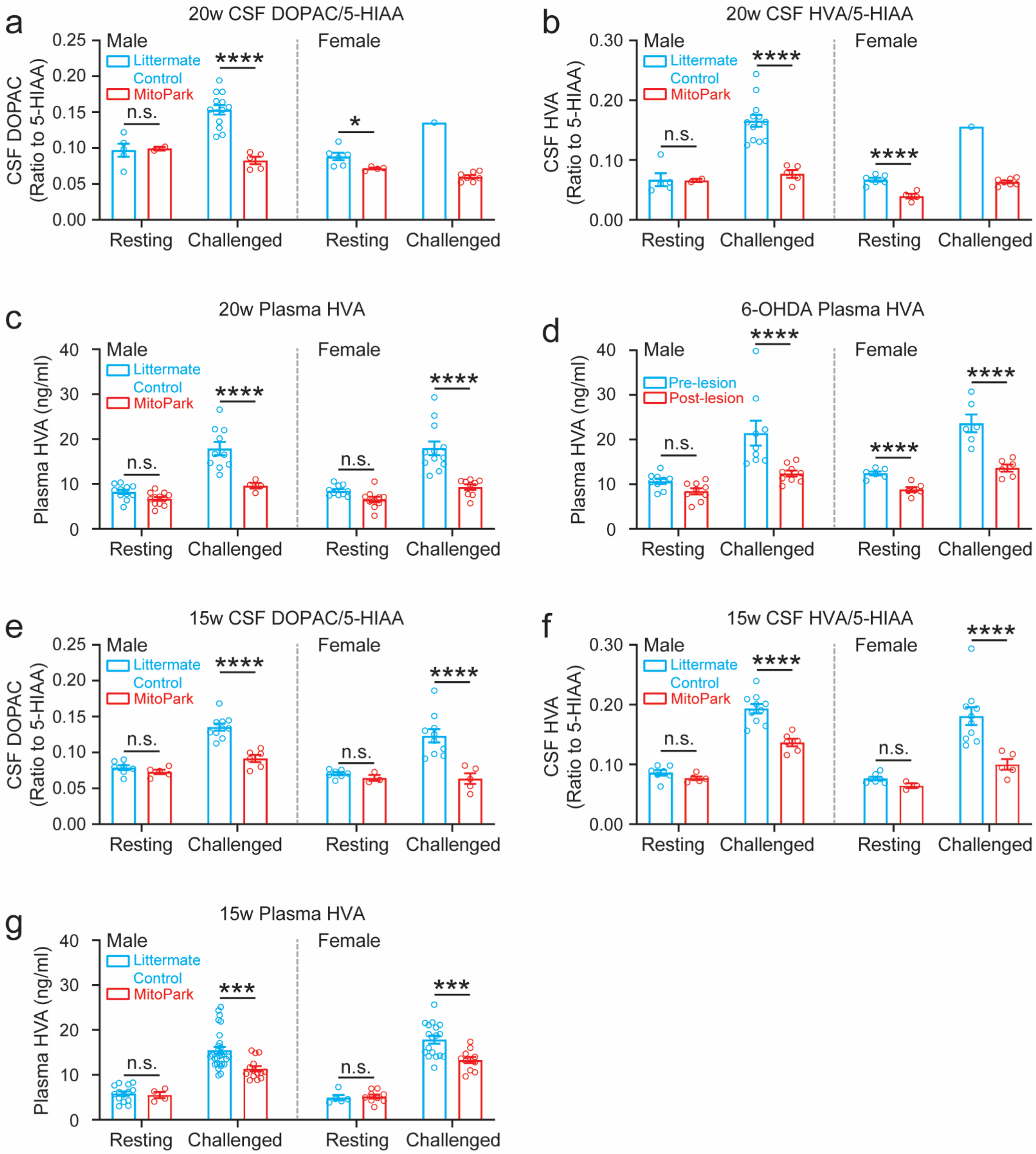

Supplementary Figure 6

Supplementary Figure 6 | Dopamine Neuron Challenge Test can detect PD in both male and female mice.

The data shown in this figure are plotted using the same set of data shown in Figure 3 and Figure 4, separating the genders.

a-c, Levels of DA metabolites in CSF (**a,b**) and plasma (**c**) at the resting state and after an i.p. injection of methylphenidate (10mg/kg) + haloperidol (1mg/kg) in 20-week-old littermate control and MitoPark mice (left, male; right, female). n.s., $p > 0.05$; *, $p < 0.05$; ****, $p < 0.0001$, two-way ANOVA followed by Sidak's multiple comparisons test. n = 5 and 2 for male control and male MitoPark mice at resting state, n = 13 and 5 for male control and male MitoPark mice after the challenge, n = 6 and 4 for female control and female MitoPark mice at resting state, n = 1 and 7 for female control and female MitoPark mice after the challenge in **a** and **b**; n = 11 and 12 for male control and male MitoPark mice at resting state, n = 10 and 4 for male control and male MitoPark mice, n = 10 and 10 for female control and female MitoPark mice at resting state, n = 12 and 9 for female control and female MitoPark mice after the challenge in **c**.

d, Plasma HVA level at the resting state and after an i.p. injection of methylphenidate (10mg/kg) + haloperidol (1mg/kg) in C57BL/6J mice before and after the unilateral 6-OHDA lesion (left, male; right, female). n.s., $p > 0.05$; ****, $p < 0.0001$, two-way ANOVA followed by Sidak's multiple comparisons test. n = 9 male and 6 female for all groups of values. All data are plotted as Mean ±SEM overlaid with individual replicates.

e-g, Levels of DA metabolites in CSF (**e,f**) and plasma (**g**) at the resting state and after an i.p. injection of methylphenidate (10mg/kg) + haloperidol (1mg/kg) in 15-week-old littermate control and MitoPark mice (left, male; right, female). n.s., $p > 0.05$; ***, $p < 0.001$; ****, $p < 0.0001$, two-way ANOVA followed by Sidak's multiple comparisons test. n = 7 and 5 for male control and male MitoPark mice at resting state, n = 10 and 6 for male control and male MitoPark mice after the challenge, n = 7 and 3 for female control and female MitoPark mice at resting state, n = 10 and 5 for female control and female MitoPark mice after the challenge in **e** and **f**; n = 14 and 4 for male control and male MitoPark at resting state, n = 29 and 14 for male control and male MitoPark, n = 5 and 9 for female control and female MitoPark at resting state, n = 18 and 11 for female control and female MitoPark after the challenge in **g**.
