# Supplementary material for: Dopamine Neuron Challenge Test for early detection of Parkinson’s disease
Source: NPJ Parkinsons Dis. 2021 Dec 16;7:116. doi: 10.1038/s41531-021-00261-z (PMC8677804; doi:10.1038/s41531-021-00261-z)
Supplement: Supplementary file 2 — Reporting Summary [file 41531_2021_261_MOESM2_ESM.pdf]

nature research

Corresponding author(s): Guohong Cui

Last updated by author(s): Oct 22, 2021

Reporting Summary

Nature Research wishes to improve the reproducibility of the work that we publish. This form provides structure for consistency and transparency in reporting. For further information on Nature Research policies, see our Editorial Policies and the Editorial Policy Checklist.

Statistics

For all statistical analyses, confirm that the following items are present in the figure legend, table legend, main text, or Methods section.

n/a	Confirmed	
☐	☒	The exact sample size (*n*) for each experimental group/condition, given as a discrete number and unit of measurement
☐	☒	A statement on whether measurements were taken from distinct samples or whether the same sample was measured repeatedly
☐	☒	The statistical test(s) used AND whether they are one- or two-sided<br>*Only common tests should be described solely by name; describe more complex techniques in the Methods section.*
☒	☐	A description of all covariates tested
☐	☒	A description of any assumptions or corrections, such as tests of normality and adjustment for multiple comparisons
☐	☒	A full description of the statistical parameters including central tendency (e.g. means) or other basic estimates (e.g. regression coefficient) AND variation (e.g. standard deviation) or associated estimates of uncertainty (e.g. confidence intervals)
☐	☒	For null hypothesis testing, the test statistic (e.g. *F*, *t*, *r*) with confidence intervals, effect sizes, degrees of freedom and *P* value noted<br>*Give P values as exact values whenever suitable.*
☒	☐	For Bayesian analysis, information on the choice of priors and Markov chain Monte Carlo settings
☒	☐	For hierarchical and complex designs, identification of the appropriate level for tests and full reporting of outcomes
☒	☐	Estimates of effect sizes (e.g. Cohen's *d*, Pearson's *r*), indicating how they were calculated

Our web collection on statistics for biologists contains articles on many of the points above.

Software and code

Policy information about availability of computer code

Data collection	1. OceanView, the software of QE-PRO spectrometer (Ocean Optics)<br>2. Chromeleon 7, the software of Dionex UltiMate 3000 UHPLC system (Thermo Scientific)<br>3. ZEN, the software of Axio observer Z1 fluorescent microscope (Carl Zeiss)<br>4. Aperio ImageScope DX, the software of Aperio AT2 slide scanner (Leica Biosystems)
Data analysis	1. Image J (NIH)<br>2. Prism (GraphPad)<br>3. A linear unmixing algorithm (https://www.niehs.nih.gov/research/atniehs/labs/ln/pi/iv/tools/index.cfm).

For manuscripts utilizing custom algorithms or software that are central to the research but not yet described in published literature, software must be made available to editors and reviewers. We strongly encourage code deposition in a community repository (e.g. GitHub). See the Nature Research guidelines for submitting code & software for further information.

Data

Policy information about availability of data

All manuscripts must include a data availability statement. This statement should provide the following information, where applicable:

- Accession codes, unique identifiers, or web links for publicly available datasets
- A list of figures that have associated raw data
- A description of any restrictions on data availability

The data that support the findings of this study are available from the corresponding authors upon request.

Field-specific reporting

Please select the one below that is the best fit for your research. If you are not sure, read the appropriate sections before making your selection.

☒ Life sciences ☐ Behavioural & social sciences ☐ Ecological, evolutionary & environmental sciences

For a reference copy of the document with all sections, see nature.com/documents/nr-reporting-summary-flat.pdf

Life sciences study design

All studies must disclose on these points even when the disclosure is negative.

Sample size	No statistical methods were used to predetermine sample size. We used sample sizes similar to the literatures in the field.
Data exclusions	No data were excluded from the analysis.
Replication	All attempts at replication were successful.
Randomization	Animals were randomly allocated into experimental groups.
Blinding	The investigators were blinded to group allocation during data collection and analysis.

Reporting for specific materials, systems and methods

We require information from authors about some types of materials, experimental systems and methods used in many studies. Here, indicate whether each material, system or method listed is relevant to your study. If you are not sure if a list item applies to your research, read the appropriate section before selecting a response.

Materials & experimental systems

n/a	Involved in the study	
☐	☒	Antibodies
☒	☐	Eukaryotic cell lines
☒	☐	Palaeontology and archaeology
☐	☒	Animals and other organisms
☒	☐	Human research participants
☒	☐	Clinical data
☒	☐	Dual use research of concern

Methods

n/a	Involved in the study	
☒	☐	ChIP-seq
☒	☐	Flow cytometry
☒	☐	MRI-based neuroimaging

Antibodies

Antibodies used

Primary antibody:
(1) Rabbit anti-TH (1:4000, ab152, Millipore/Chemicon)
(2) Chicken anti-GFP (1:1000, ab13970, Abcam)

Secondary antibody:
(1) Alexa Fluor 488 conjugated goat anti-chicken (1:500, A-11039, Invitrogen)
(2) Alexa Fluor 647 conjugated goat anti rabbit (1:1000, A-21245, Invitrogen)

Validation

All antibodies were validated either by the manufacturer or used extensively in published research articles.

Rabbit anti-TH (1:4000, ab152, Millipore/Chemicon)(The manufacturer website states that this antibody has been validated with mouse brain crysection by Immunocytochemistry)
Chicken anti-GFP (1:1000, ab13970, Abcam)(The manufacturer website states that this antibody has been validated with Adenoviral Cre induces mosaic activation of rtTA and GFP induction in CAGs-LSL-rtTA3 and CAGs-LSL-RIK animals by Immunocytochemistry)

Alexa Fluor 488 conjugated goat anti-chicken (1:500, A-11039, Invitrogen)(The manufacturer website states that this antibody has been validated with mouse intestine cryosection showing basement membranes labeled with chicken IgY anti-fibronectin antibody by Immunocytochemistry)
lexa Fluor 647 conjugated goat anti rabbit (1:1000, A-21245, Invitrogen)(The manufacturer website states that this antibody has been validated with HeLa cells stained with alpha Tubulin Rabbit Polyclonal Antibody by Immunocytochemistry)

Animals and other organisms

Policy information about studies involving animals; ARRIVE guidelines recommended for reporting animal research

Laboratory animals	1. Adult (P56–280) wild-type C57BL/6J (Jackson Laboratory, Stock# 000664), male and female.<br>2. Adult (P56–140) DATIREScre/+;Tfamflox/+ in house bred by crossing DATIREScre/+ mice21 (006660, Jackson Laboratory) with Tfamflox/flox (026123, Jackson Laboratory), male and female.<br>3. Adult (P56–140) Tfamflox/flox in house bred by crossing DATIREScre/+;Tfamflox/+ (in house bred) with Tfamflox/flox (026123, Jackson Laboratory), male and female.<br>4. Adult (P56–140) Tfamflox/+ in house bred by crossing DATIREScre/+;Tfamflox/+ (in house bred) with Tfamflox/flox (026123, Jackson Laboratory), male and female.<br>5. Adult (P56–140) DATIREScre/+;Tfamflox/flox in house bred by crossing DATIREScre/+;Tfamflox/+ (in house bred) with Tfamflox/flox (026123, Jackson Laboratory), male and female.
Wild animals	This study did not involve wild animals.
Field-collected samples	This study did not involve samples collected from the fields.
Ethics oversight	All procedures for animal surgery, maintenance, and behavior were performed using protocols that were approved by the animal care and use committees at the US National Institutes of Health.

Note that full information on the approval of the study protocol must also be provided in the manuscript.
